# Supplementary material for: Dexamethasone and OLT1177 Cooperate in the Reduction of Melanoma Growth by Inhibiting STAT3 Functions
Source: Cells. 2023 Jan 12;12(2):294. doi: 10.3390/cells12020294 (PMC9856388; doi:10.3390/cells12020294)
Supplement: Supplementary file 1 [file cells-12-00294-s001.zip › cells-2158144-supplementary.pdf]

Supplementary Table S1. List of primers (5′-3′ sequences) used for RT-qPCR.

Gene	Forward sequence	Reverse sequence	Species
Socs3	ATTTCGCTTCGGGACTAGC	AACTTGCTGTGGGTGACCAT	Mouse
Mt-nd1	CCATTCTAATCGCCATAGCC	ATGCCGTATGGACCAACAAT	Mouse
Mt-nd4	CGCCTACTCCTCAGTTAGCC	GTGAGGCCATGTGCGATTAT	Mouse
Klf4	CGGGAAGGGAGAAGACACT	GAGTTCCTCACGCCAACG	Mouse
Fkbp5	GGAAGGAGAAGACCACGACA	ACATAAGCTCAGCATTGGGG	Mouse
Hif1a	CTATGGAGGCCAGAAGAGGGTAT	CCCACATCAGGTGGCTCATAA	Mouse
Vcam	TGGGAGAGACAAAGCAGAAGT	TCAGAACAACCGAATCCCCA	Mouse
Icam	TGCCTCTGAAGCTCGGATATA	TCTGTCGAACTCCTCAGTCAC	Mouse
Vegf	GCATTCACATCTGCTGTGCT	CAGGCTGCTGTAACGATGAA	Mouse
18S	GTAACCCGTTGAACCCCATT	CCATCCAATCGGTAGTAGCG	Mouse
SOCS3	GGCCACTCTTCAGCATCTCT	TTAAAGCGGGGCATCGTACT	Human
MT-ND1	CCCCTTCGCCCTATTCTTCA	AGGGGAGAGTGCGTCATATG	Human
MT-ND4	CTCACTCTCACTGCCCAAGA	GCTTCGACATGGGCTTTAGG	Human
HK1	TCCAACAGCCACAGTCAAGA	AGGAAGACCCACCAAGATCC	Human
HK2	AGCCACCACTCACCCTAC	AAAGCACACGGAAGTTGGTC	Human
PFKP	TGCTCCTTCAGCCACAATAA	GACTGGGTGTTCCTTCCAGA	Human

PPARGC1A	GAGAGTCTGAGAGGGCCAAG	GTTCTGTCCGTGTTGTGTCA	Human
PPARGC1B	GGTGGACGAGCTCTCACTG	CTTTTAGATCTGAGGCCTGCC	Human
TFAM	TGGTTTTCATCTGTCTTGGCA	GAAGTTCCCTCCAACGCTG	Human
MFN1	CAGCAGTGAAAACAAAGTTCGA	TCTTCAGCTGCCAGGTTTAC	Human
MFN2	GAAGACCAAGTTTGAGCAGCA	ATAAATTTCAGTCGGTCTTGCC	Human
OPA1	TGGAAAGATATGATACCGGACCT	CTTGTCAAAGTCTGGTGCTAACT	Human
UCP2	GGACCTCTCCCAATGTTGCT	CGATGACAGTGGTGCAGAAG	Human
UCP3	CCTCCAGGCCAGTACTTCAG	CTTTCATCAGGGCCCGTTTC	Human
ACTB	CTCCATCCTGGCCTCGCTGT	GCTGTCACCTTCACCGTTCC	Human

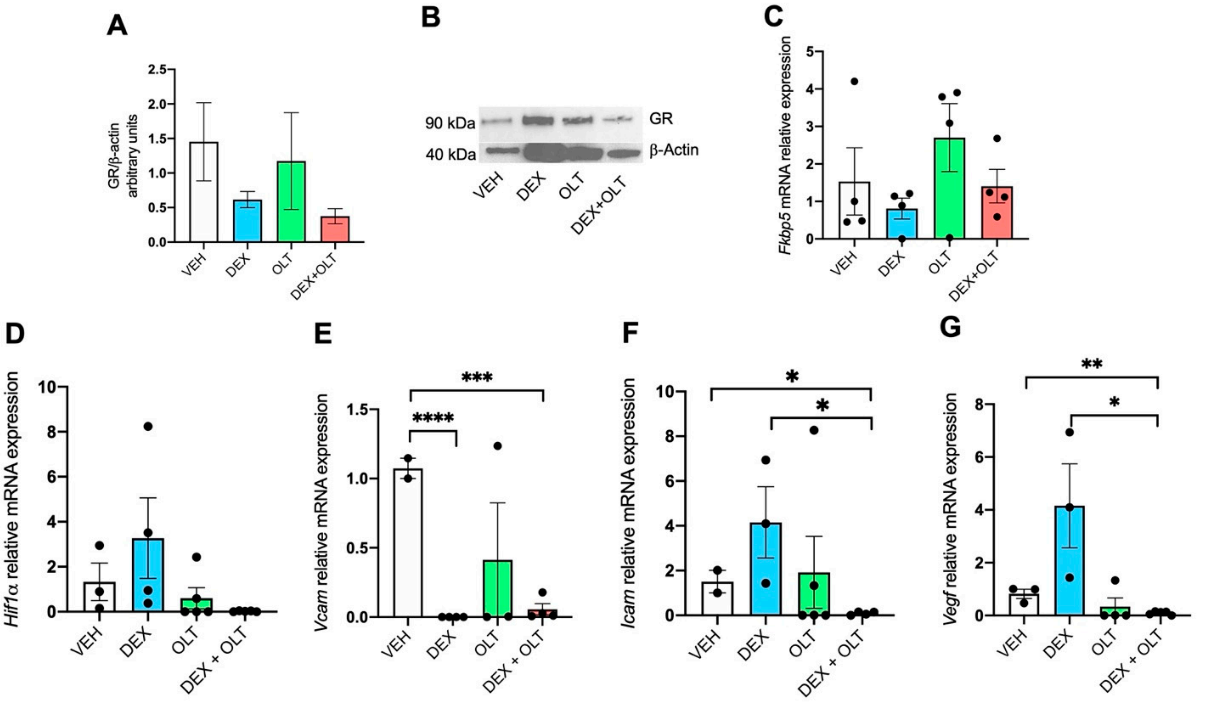

Figure S1. Expression levels of protumor marker genes. (**A**) A representative western blot performed on primary tumor samples. (**B**) Histogram derived from western blot (shown in A) of GR levels in the primary tumor. (**C**–**G**) Expression levels in primary tumors of (C) *Fkbp5*, (**D**) *Hif1a*, (E) *Vcam*, (F) *Icam*, and (G) Vegf. The data are presented as mean ± SEM. *p<0.05, **p<0.01, ***p<0.001, ****p<0.0001.

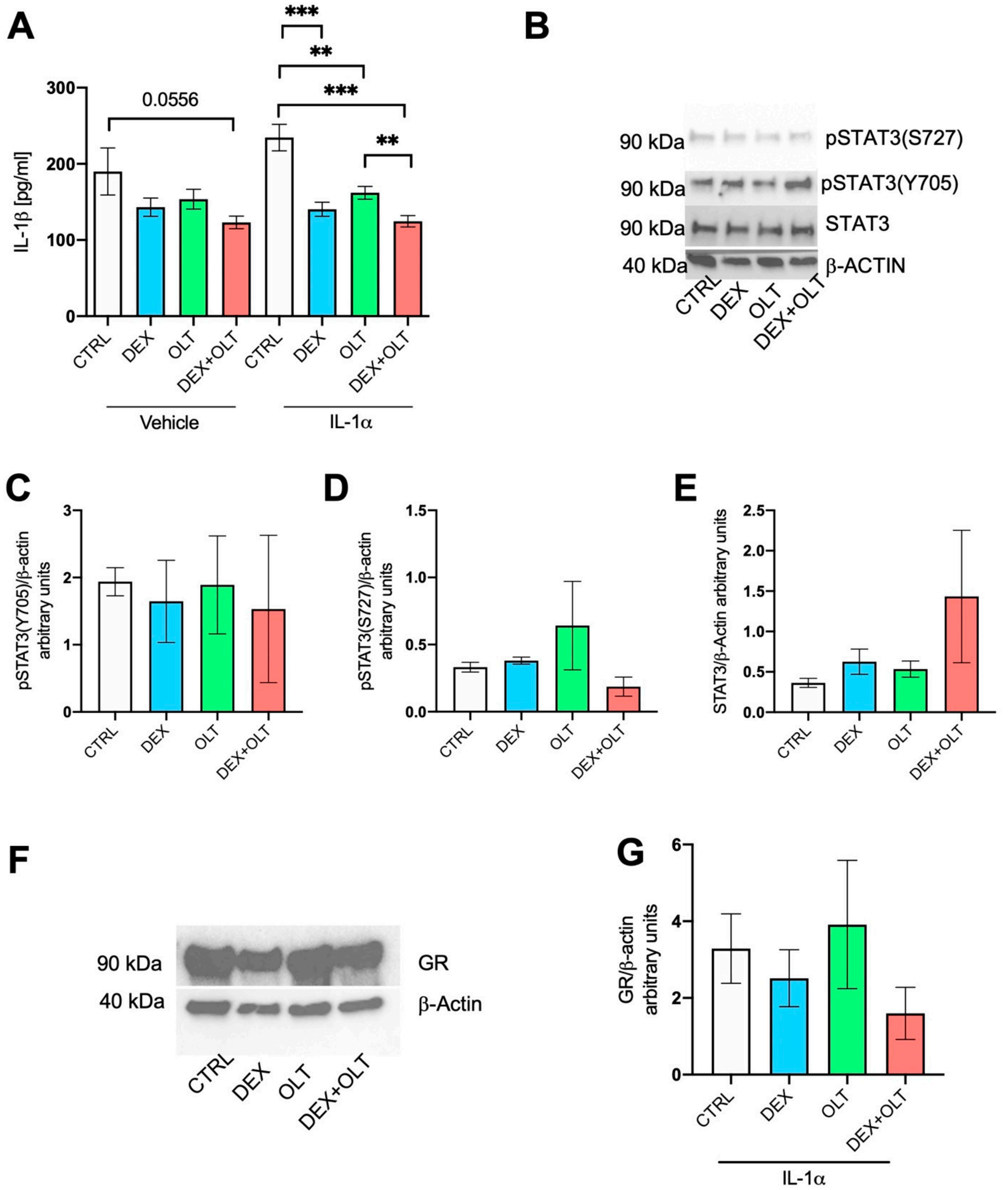

Figure S2. Effects of treatments on IL-1β, STAT3 and GR in 1205Lu cells (**A**) IL-1β levels in supernatants of 1205Lu c cells stimulated with IL-1α or vehicle and then treated with IL-1α, dexamethasone, OLT1177 or dexamethasone plus OLT1177. (**B**) A representative western blot performed on primary 1205Lu cells. (**C**) Histogram derived from western blot (shown in B) of pSTAT3 Y705 levels in 1205Lu cells. (**D**) Histogram derived from western blot (shown in B) of pSTAT3 S727 levels in 1205Lu cells. (**E**) Histogram derived from western blot (shown in B) of STAT3 levels in 1205Lu cells. (**F**) A representative western blot performed on primary 1205Lu cells treated with IL-1α. (**G**) Histogram derived from western blot (shown in F) of GR levels in 1205Lu cells. The data are presented as mean ± SEM. **$p<0.01$; ***$p<0.001$.

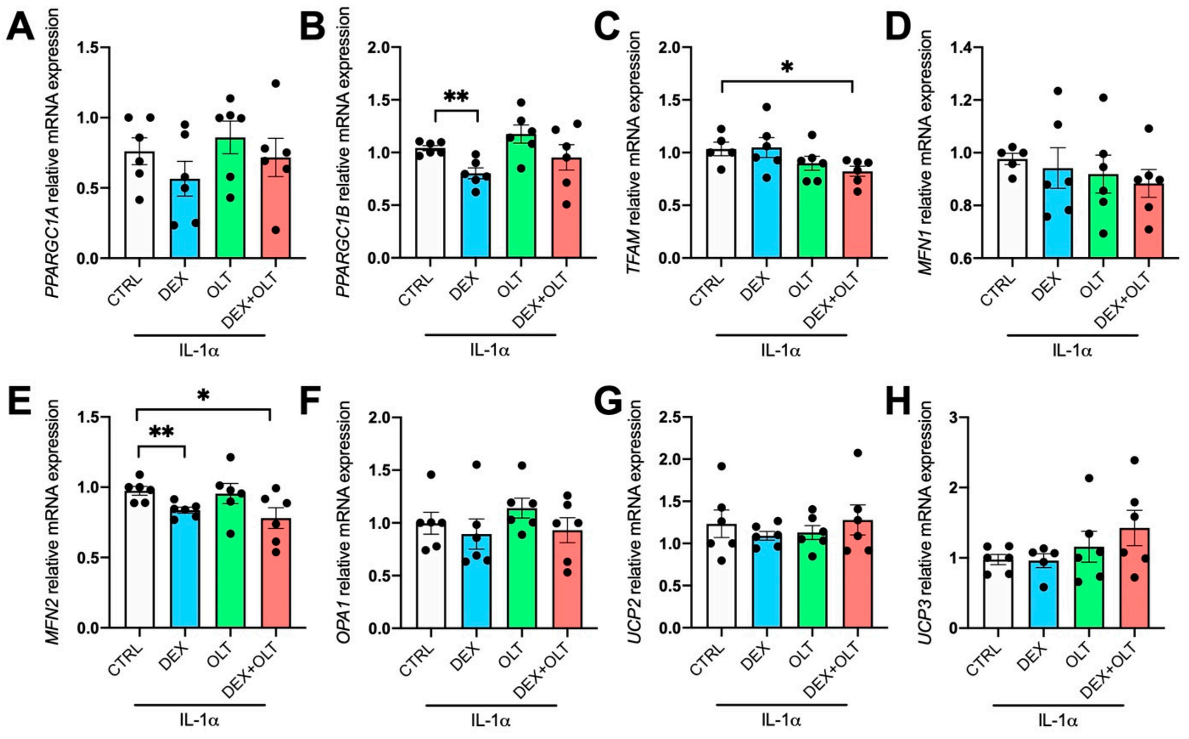

Figure S3. Analysis of mitochondria-related genes in 1205Lu cells. (**A-H**) Gene expression of (**A**) *PPARGC1A*, (**B**) *PPARGC1B*, (**C**) *TFAM*, (**D**) *MFN1*, (**E**) *MFN2*, (**F**) *OPA1*, (**G**) *UCP2*, and (**H**) *UCP3* in 1205Lu cells stimulated with IL-1α and then with IL-1α, dexamethasone, OLT1177 or dexamethasone plus OLT1177. The data are presented as mean ± SEM. *p<0.05; **p<0.01.

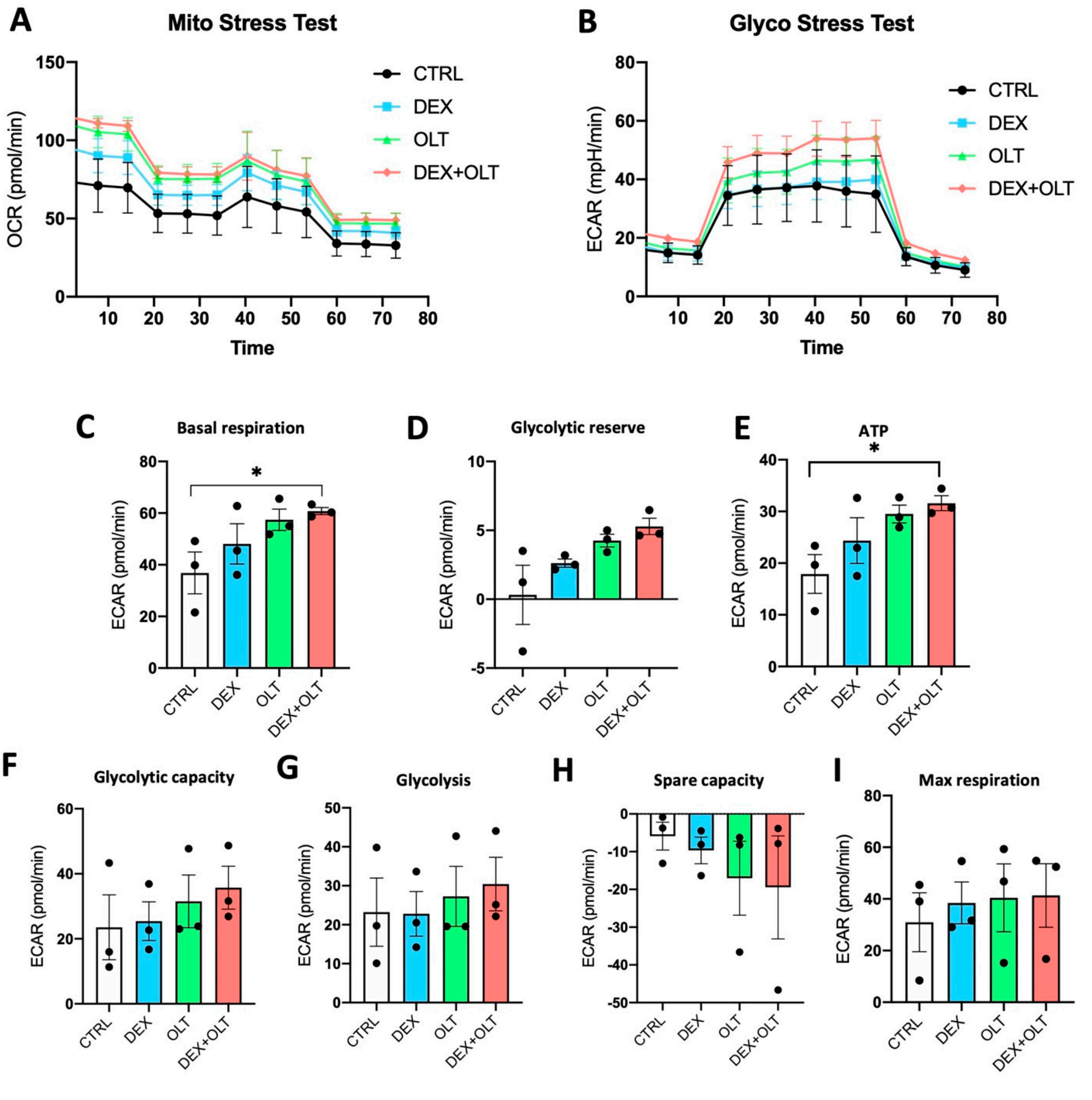

Figure S4. Dexamethasone and OLT1177 effects on OCR and ECAR of 1205Lu cells. (**A**) Measurement of OCR in 1205Lu treated with vehicle, dexamethasone, OLT1177 or the combination of dexamethasone plus OLT1177 (Mito stress test). (**B**) Measurement of ECAR in LU1205 treated vehicle, dexamethasone, OLT1177 and dexamethasone+OLT1177 (Glyco stress test). (**C**) Basal respiration rate measured in control 1205Lu treated vehicle, dexamethasone, OLT1177, dexamethasone+OLT1177 for 16 hours. (**D**) Glycolytic reserve rate measured in control 1205Lu treated with vehicle, dexamethasone, OLT1177, dexamethasone+OLT1177 for 16 hours. (**E**) ATP rate measured in control 1205Lu treated with vehicle, dexamethasone, OLT1177, dexamethasone+OLT1177 for 16 hours. (**F**) Glycolytic capacity rate measured in control 1205Lu treated with vehicle, dexamethasone, OLT1177, dexamethasone+OLT1177 for 16 hours. (**G**) Glycolysis rate measured in control 1205Lu treated with vehicle, dexamethasone, OLT1177, dexamethasone+OLT1177 for 16 hours. (**H**) Spare capacity rate measured in control 1205Lu treated with vehicle, dexamethasone, OLT1177, dexamethasone+OLT1177 for 16 hours. (**I**) Maximal respiration rate measured in control 1205Lu treated with vehicle, dexamethasone, OLT1177, dexamethasone+OLT1177 for 16 hours. The data are presented as mean ± SEM. *p<0.05.

Supplementary Table S2. Statistical analysis of Figure S4A.

Tukey's multiple comparisons test	Mean Diff.	95.00% CI of diff.	Significant?	Summary	Adjusted P Value
Row 1					
Con vs. Dex	-21.63	-60.98 to 17.71	No	ns	0.4792
Con vs. OLT	-36.64	-75.98 to 2.702	No	ns	0.0773
Con vs. Combo	-41.47	-80.81 to -2.125	Yes	*	0.0348
Dex vs. OLT	-15.01	-54.35 to 24.34	No	ns	0.7513
Dex vs. Combo	-19.83	-59.18 to 19.51	No	ns	0.5538
OLT vs. Combo	-4.827	-44.17 to 34.52	No	ns	0.9885
Row 2					
Con vs. Dex	-19.04	-58.39 to 20.30	No	ns	0.5869
Con vs. OLT	-34.22	-73.56 to 5.122	No	ns	0.1113
Con vs. Combo	-39.83	-79.17 to -0.4848	Yes	*	0.0461
Dex vs. OLT	-15.18	-54.52 to 24.17	No	ns	0.7448
Dex vs. Combo	-20.78	-60.13 to 18.56	No	ns	0.5141
OLT vs. Combo	-5.607	-44.95 to 33.74	No	ns	0.9822
Row 3					
Con vs. Dex	-19.27	-58.61 to 20.08	No	ns	0.5776
Con vs. OLT	-34.20	-73.54 to 5.145	No	ns	0.1117
Con vs. Combo	-39.58	-78.92 to -0.2348	Yes	*	0.0481
Dex vs. OLT	-14.93	-54.27 to 24.41	No	ns	0.7542
Dex vs. Combo	-20.31	-59.65 to 19.03	No	ns	0.5338
OLT vs. Combo	-5.380	-44.72 to 33.96	No	ns	0.9842
Row 4					
Con vs. Dex	-11.90	-51.24 to 27.45	No	ns	0.8585
Con vs. OLT	-22.01	-61.35 to 17.34	No	ns	0.4641
Con vs. Combo	-26.01	-65.35 to 13.34	No	ns	0.3148
Dex vs. OLT	-10.11	-49.45 to 29.23	No	ns	0.9075
Dex vs. Combo	-14.11	-53.45 to 25.23	No	ns	0.7846
OLT vs. Combo	-4.000	-43.34 to 35.34	No	ns	0.9934
Row 5					
Con vs. Dex	-11.77	-51.11 to 27.57	No	ns	0.8623
Con vs. OLT	-22.04	-61.38 to 17.30	No	ns	0.4627
Con vs. Combo	-25.27	-64.61 to 14.07	No	ns	0.3402
Dex vs. OLT	-10.27	-49.61 to 29.07	No	ns	0.9035
Dex vs. Combo	-13.50	-52.84 to 25.84	No	ns	0.8063
OLT vs. Combo	-3.230	-42.57 to 36.11	No	ns	0.9965
Row 6					
Con vs. Dex	-13.16	-52.50 to 26.19	No	ns	0.8181
Con vs. OLT	-23.52	-62.86 to 15.83	No	ns	0.4046
Con vs. Combo	-26.31	-65.65 to 13.04	No	ns	0.3048
Dex vs. OLT	-10.36	-49.70 to 28.98	No	ns	0.9013
Dex vs. Combo	-13.15	-52.49 to 26.19	No	ns	0.8183
OLT vs. Combo	-2.790	-42.13 to 36.55	No	ns	0.9977
Row 7					
Con vs. Dex	-15.51	-54.85 to 23.83	No	ns	0.7319
Con vs. OLT	-23.07	-62.41 to 16.28	No	ns	0.4220
Con vs. Combo	-26.01	-65.36 to 13.33	No	ns	0.3146
Dex vs. OLT	-7.557	-46.90 to 31.79	No	ns	0.9584
Dex vs. Combo	-10.50	-49.85 to 28.84	No	ns	0.8976
OLT vs. Combo	-2.947	-42.29 to 36.40	No	ns	0.9973
Row 8					
Con vs. Dex	-13.11	-52.45 to 26.24	No	ns	0.8198
Con vs. OLT	-19.70	-59.05 to 19.64	No	ns	0.5592
Con vs. Combo	-22.95	-62.29 to 16.39	No	ns	0.4266
Dex vs. OLT	-6.597	-45.94 to 32.75	No	ns	0.9717
Dex vs. Combo	-9.843	-49.19 to 29.50	No	ns	0.9138
OLT vs. Combo	-3.247	-42.59 to 36.10	No	ns	0.9964
Row 9					
Con vs. Dex	-12.76	-52.11 to 26.58	No	ns	0.8312
Con vs. OLT	-19.34	-58.68 to 20.00	No	ns	0.5745
Con vs. Combo	-23.04	-62.39 to 16.30	No	ns	0.4229
Dex vs. OLT	-6.577	-45.92 to 32.77	No	ns	0.9719
Dex vs. Combo	-10.28	-49.62 to 29.06	No	ns	0.9033
OLT vs. Combo	-3.703	-43.05 to 35.64	No	ns	0.9947
Row 10					
Con vs. Dex	-7.983	-47.33 to 31.36	No	ns	0.9514
Con vs. OLT	-12.95	-52.30 to 26.39	No	ns	0.8249
Con vs. Combo	-15.02	-54.37 to 24.32	No	ns	0.7506
Dex vs. OLT	-4.970	-44.31 to 34.37	No	ns	0.9875
Dex vs. Combo	-7.040	-46.38 to 32.30	No	ns	0.9659
OLT vs. Combo	-2.070	-41.41 to 37.27	No	ns	0.9991
Row 11					
Con vs. Dex	-8.323	-47.67 to 31.02	No	ns	0.9455
Con vs. OLT	-13.10	-52.44 to 26.24	No	ns	0.8200
Con vs. Combo	-15.69	-55.03 to 23.65	No	ns	0.7248
Dex vs. OLT	-4.777	-44.12 to 34.57	No	ns	0.9889
Dex vs. Combo	-7.367	-46.71 to 31.98	No	ns	0.9612
OLT vs. Combo	-2.590	-41.93 to 36.75	No	ns	0.9982
Row 12					
Con vs. Dex	-7.977	-47.32 to 31.37	No	ns	0.9516
Con vs. OLT	-13.86	-53.21 to 25.48	No	ns	0.7935
Con vs. Combo	-16.14	-55.49 to 23.20	No	ns	0.7068
Dex vs. OLT	-5.887	-45.23 to 33.46	No	ns	0.9796
Dex vs. Combo	-8.167	-47.51 to 31.18	No	ns	0.9483
OLT vs. Combo	-2.280	-41.62 to 37.06	No	ns	0.9988

Supplementary Table S3. Statistical analysis of Figure S4B.

Tukey's multiple comparisons test	Mean Diff.	95.00% CI of diff.	Significant?	Summary	Adjusted P Value
Row 1					
Con vs. Dex	-1.280	-23.61 to 21.05	No	ns	0.9988
Con vs. OLT	-2.603	-24.93 to 19.72	No	ns	0.9901
Con vs. Combo	-5.560	-27.89 to 16.77	No	ns	0.9149
Dex vs. OLT	-1.323	-23.65 to 21.00	No	ns	0.9987
Dex vs. Combo	-4.280	-26.61 to 18.05	No	ns	0.9586
OLT vs. Combo	-2.957	-25.28 to 19.37	No	ns	0.9857
Row 2					
Con vs. Dex	-0.04333	-22.37 to 22.28	No	ns	>0.9999
Con vs. OLT	-1.543	-23.87 to 20.78	No	ns	0.9979
Con vs. Combo	-4.920	-27.25 to 17.41	No	ns	0.9390
Dex vs. OLT	-1.500	-23.83 to 20.83	No	ns	0.9981
Dex vs. Combo	-4.877	-27.20 to 17.45	No	ns	0.9405
OLT vs. Combo	-3.377	-25.70 to 18.95	No	ns	0.9789
Row 3					
Con vs. Dex	-0.3300	-22.66 to 22.00	No	ns	>0.9999
Con vs. OLT	-1.503	-23.83 to 20.82	No	ns	0.9980
Con vs. Combo	-4.460	-26.79 to 17.87	No	ns	0.9535
Dex vs. OLT	-1.173	-23.50 to 21.15	No	ns	0.9991
Dex vs. Combo	-4.130	-26.46 to 18.20	No	ns	0.9626
OLT vs. Combo	-2.957	-25.28 to 19.37	No	ns	0.9857
Row 4					
Con vs. Dex	-0.2000	-22.53 to 22.13	No	ns	>0.9999
Con vs. OLT	-5.147	-27.47 to 17.18	No	ns	0.9310
Con vs. Combo	-11.34	-33.67 to 10.99	No	ns	0.5475
Dex vs. OLT	-4.947	-27.27 to 17.38	No	ns	0.9381
Dex vs. Combo	-11.14	-33.47 to 11.19	No	ns	0.5623
OLT vs. Combo	-6.193	-28.52 to 16.13	No	ns	0.8868
Row 5					
Con vs. Dex	-0.2867	-22.61 to 22.04	No	ns	>0.9999
Con vs. OLT	-5.780	-28.11 to 16.55	No	ns	0.9056
Con vs. Combo	-12.45	-34.78 to 9.874	No	ns	0.4666
Dex vs. OLT	-5.493	-27.82 to 16.83	No	ns	0.9176
Dex vs. Combo	-12.17	-34.49 to 10.16	No	ns	0.4871
OLT vs. Combo	-6.673	-29.00 to 15.65	No	ns	0.8627
Row 6					
Con vs. Dex	-0.09333	-22.42 to 22.23	No	ns	>0.9999
Con vs. OLT	-5.467	-27.79 to 16.86	No	ns	0.9187
Con vs. Combo	-11.74	-34.06 to 10.59	No	ns	0.5184
Dex vs. OLT	-5.373	-27.70 to 16.95	No	ns	0.9224
Dex vs. Combo	-11.64	-33.97 to 10.68	No	ns	0.5252
OLT vs. Combo	-6.270	-28.60 to 16.06	No	ns	0.8831
Row 7					
Con vs. Dex	-1.390	-23.72 to 20.94	No	ns	0.9985
Con vs. OLT	-8.663	-30.99 to 13.66	No	ns	0.7414
Con vs. Combo	-16.17	-38.50 to 6.157	No	ns	0.2376
Dex vs. OLT	-7.273	-29.60 to 15.05	No	ns	0.8295
Dex vs. Combo	-14.78	-37.11 to 7.547	No	ns	0.3135
OLT vs. Combo	-7.507	-29.83 to 14.82	No	ns	0.8157
Row 8					
Con vs. Dex	-3.193	-25.52 to 19.13	No	ns	0.9821
Con vs. OLT	-10.22	-32.55 to 12.10	No	ns	0.6301
Con vs. Combo	-17.57	-39.90 to 4.757	No	ns	0.1746
Dex vs. OLT	-7.030	-29.36 to 15.30	No	ns	0.8433
Dex vs. Combo	-14.38	-36.70 to 7.950	No	ns	0.3380
OLT vs. Combo	-7.347	-29.67 to 14.98	No	ns	0.8252
Row 9					
Con vs. Dex	-4.963	-27.29 to 17.36	No	ns	0.9375
Con vs. OLT	-11.81	-34.13 to 10.52	No	ns	0.5133
Con vs. Combo	-19.06	-41.38 to 3.270	No	ns	0.1221
Dex vs. OLT	-6.843	-29.17 to 15.48	No	ns	0.8536
Dex vs. Combo	-14.09	-36.42 to 8.234	No	ns	0.3557
OLT vs. Combo	-7.250	-29.58 to 15.08	No	ns	0.8308
Row 10					
Con vs. Dex	-0.1900	-22.52 to 22.14	No	ns	>0.9999
Con vs. OLT	-1.230	-23.56 to 21.10	No	ns	0.9989
Con vs. Combo	-4.677	-27.00 to 17.65	No	ns	0.9470
Dex vs. OLT	-1.040	-23.37 to 21.29	No	ns	0.9993
Dex vs. Combo	-4.487	-26.81 to 17.84	No	ns	0.9528
OLT vs. Combo	-3.447	-25.77 to 18.88	No	ns	0.9776
Row 11					
Con vs. Dex	-0.6033	-22.93 to 21.72	No	ns	0.9999
Con vs. OLT	-1.533	-23.86 to 20.79	No	ns	0.9979
Con vs. Combo	-4.073	-26.40 to 18.25	No	ns	0.9640
Dex vs. OLT	-0.9300	-23.26 to 21.40	No	ns	0.9995
Dex vs. Combo	-3.470	-25.80 to 18.86	No	ns	0.9772
OLT vs. Combo	-2.540	-24.87 to 19.79	No	ns	0.9908
Row 12					
Con vs. Dex	-0.5833	-22.91 to 21.74	No	ns	0.9999
Con vs. OLT	-1.167	-23.49 to 21.16	No	ns	0.9991
Con vs. Combo	-3.393	-25.72 to 18.93	No	ns	0.9786
Dex vs. OLT	-0.5833	-22.91 to 21.74	No	ns	0.9999
Dex vs. Combo	-2.810	-25.14 to 19.52	No	ns	0.9876
OLT vs. Combo	-2.227	-24.55 to 20.10	No	ns	0.9937
